# College and Hospital Notes

**Published:** 1908-06

**Authors:** 


### COLLEGE AND HOSPITAL NOTES.

---

The sixty-third annual commencement exercises of the University of Buffalo, including the departments of medicine, law, pharmacy and dentistry, were held at the Teck theater Friday, May 29, 1908, at 11 A. M. The address was delivered by the Rev. S. Van Vranken Holmes, D.D. The faculty of medicine held a reception afterward to the graduates, their friends, and the alumni at the University club, during which a luncheon was served.

Beginning on Tuesday, May 26, the Alumni Association held its annual meeting and during the next two days, May 27, and 28, a series of clinics were held at the various hospitals. Allusion to these meetings and clinics was made in this column for May, and in the July issue we expect to publish a detailed account of the proceedings for the week. This edition of the Journal goes to press too early to publish the proceedings in the June number.

---

The United States Steamship Buffalo is to become a hospital ship. With a view to preparedness in case of necessity, the surgeon general's office of the navy has suggested the designation of several vessels available for use as hospital ships. Among the ships suggested by Surgeon General Rixey is the Buffalo, now on the Pacific Coast, which is regarded by naval surgeons as admirably suited for such purposes. The surgeon general is an earnest advocate of the use of hospital ships and takes the ground that the navy should always be ready with them if a call is made for their services.

---

The Lockport common council, at a recent meeting, officially accepted the gift of $10 000 from the estate of Morrison W. Evans for the construction of an addition to the new hospital. The annex will be known as the Morrison W. and Agnes H. Evans Memorial, and must be under way within three months and completed within a year. Of the fund originally left the city $1,500 has been expended for instruments for the new hospital.

